# Antibiotic Resistance Profile, Outer Membrane Proteins, Virulence Factors and Genome Sequence Analysis Reveal Clinical Isolates of *Enterobacter* Are Potential Pathogens Compared to Environmental Isolates

**DOI:** 10.3389/fcimb.2020.00054

**Published:** 2020-02-21

**Authors:** Mitali Mishra, Sasmita Panda, Susmita Barik, Arup Sarkar, Durg Vijai Singh, Harapriya Mohapatra

**Affiliations:** ^1^School of Biological Sciences, National Institute of Science Education and Research, HBNI, Bhubaneswar, India; ^2^Homi Bhabha National Institute (HBNI), Mumbai, India; ^3^Infectious Disease Biology, Institute of Life Sciences, Bhubaneswar, India; ^4^Trident School of Biotech Sciences, Trident Academy of Creative and Technology, Bhubaneswar, India

**Keywords:** aquatic environment, outer membrane proteins (OMPs), *Enterobacter* spp., association, antibiotic resistance, virulence, multidrug resistance (MDR)

## Abstract

Outer membrane proteins (OMPs) of gram-negative bacteria play an important role in mediating antibacterial resistance, bacterial virulence and thus affect pathogenic ability of the bacteria. Over the years, prevalence of environmental antibiotic resistant organisms, their transmission to clinics and ability to transfer resistance genes, have been studied extensively. Nevertheless, how successful environmental bacteria can be in establishing as pathogenic bacteria under clinical setting, is less addressed. In the present study, we utilized an integrated approach of investigating the antibiotic resistance profile, presence of outer membrane proteins and virulence factors to understand extent of threat posed due to multidrug resistant environmental *Enterobacter* isolates. Also, we investigated clinical *Enterobacter* isolates and compared the results thereof. Results of the study showed that multidrug resistant environmental *Enterobacter* isolates lacked OmpC, lacked cell invasion abilities and exhibited low reactive oxygen species (ROS) production in neutrophils. In contrast, clinical isolates possessed OmpF, exhibited high invasive and adhesive property and produced higher amounts of ROS in neutrophils. These attributes indicated limited pathogenic potential of environmental *Enterobacter* isolates. Informations obtained from whole genome sequence of two representative bacterial isolates from environment (DL4.3) and clinical sources (EspIMS6) corroborated well with the observed results. Findings of the present study are significant as it highlights limited fitness of multidrug resistant environmental *Enterobacter* isolates.

## Introduction

Development of antibiotic resistance in pathogens has emerged as global health problem. Our knowledge about the resistance mechanisms has been significantly enriched during last decade (O'Neill, 2014). Extensive research has provided credible information that have pointed toward co-evolution of antibiotic resistance in both natural environment and clinics (Davies and Davies, 2010). So far, our understanding on origin and escalation of environmental antibiotic resistance, infers that bacterial isolates possess inherent and adaptive resistance mechanisms that upon exposure to antibiotics/stimuli gets induced. Such antibiotic resistance determinants are transmitted to other strains by various means, ultimately resulting in emergence of resistant strains (Wellington et al., 2013; Bengtsson-Palme et al., 2018). Environmental antibiotic resistant bacteria (eARB) act as a reservoir of antibiotic resistance genes (ARGs), which under selective pressure could transform into pathogenic antibiotic resistant bacteria (pARB), that pose serious health risk resulting in treatment failure (Ashbolt et al., 2013).

The gram-negative, facultative anaerobic, rod-shaped bacteria belonging to Enterobacteriaceae family, *Enterobacter* is a nosocomial pathogen, having ubiquitous distribution in natural environment including sewage and dairy products (Grimont and Grimont, 2006; Davin-Regli and Pagès, 2015). These bacteria are increasingly exhibiting multidrug resistance phenotype, thus becoming resilient to available treatment therapies. The aforementioned fact has resulted in them being included in the “ESKAPE” group of opportunistic pathogens that represents a group for which in a given scenario, no effective therapeutic options would be available (Boucher et al., 2009; Rice, 2010). Also, there is escalation in reports of *Enterobacter* spp. exhibiting resistance toward the last line of antibiotics *viz*. carbapenems and colistin (Thiolas et al., 2005; Le-Ha et al., 2019). *Enterobacter cloacae* and *Enterobacter aerogenes*, are associated with a plethora of diseases such as lower respiratory tract infections, pneumonia, urinary tract infections, skin/soft-tissue infections, septicemia, wound infections, meningitis and nosocomial infections in intensive care units (Davin-Regli and Pagès, 2015).

Outer membrane of gram-negative bacteria is an asymmetric lipid bilayer that permits selective influx of solutes into the cell (Pagès et al., 2008). The outer membrane contains water filled open channels that facilitate passive penetration of hydrophilic drugs restricted to <600 kDa. The proteins that constitute these pores are generally referred to as porins (Fernández and Hancock, 2012). Based on their function and architecture, the porins or outer-membrane proteins (OMPs) are categorized into small β-barrel membrane anchors (e.g., OmpA, OmpX), general non-specific porins (e.g., OmpF, OmpC), substrate specific porins (e.g., PhoE, LamB) and TonB-dependent receptors (e.g., FhuA, FepA; Koebnik et al., 2000). Besides their roles as solute carriers, OMPs have diverse physiological roles in bacteria (Lin et al., 2002); for example, OmpX neutralizes host defense mechanisms, OmpA establishes a physical linkage between outer membrane, and peptidoglycan layer (Buchanan, 1999), OmpC and OmpF are responsible for influx of antibiotics and other solutes. While porins, like OmpA, are expressed constitutively in cells, expression of others such as LamB, PhoE, FhuA, are induced in presence of either specific substrate or by environmental stimuli (Koebnik et al., 2000). Development of multidrug resistance phenotype in such gram-negative pathogens has been associated with porin modification in three ways: by alterations in porin expression, by decreased porin expression, and by mutation in porins. All of the above aspects, individually or in combination affect bacterial susceptibility toward antibiotics, particularly the β-lactams (Pagès et al., 2008). A coordinated interplay between outer membrane protein expression and subsequent folding, increased efflux activity and controlled outer membrane permeability, have been associated with multidrug resistant (MDR) phenotype in *E. coli* (Viveiros et al., 2007). In *Acinetobacter baumannii*, OmpA disruption lead to severe reduction in minimum inhibitory concentration for multiple antibiotics, suggesting its contribution toward MDR phenotype (Smani et al., 2014).

Besides facilitating antibiotic resistance, OMPs serve as receptors for bacteriocins, hemolysin, other toxins and antibodies (Smani et al., 2014). OMPs are also believed to play a pivotal role in bacterial pathogenesis. OmpA, facilitates bacterial adhesion to mucosal surfaces, invasion, serum resistance and antimicrobial peptide resistance (Confer and Ayalew, 2013). In *Cronobacter sakazakii*, compared to the wild type isolates, deletion mutants of OmpA and OmpX isolates exhibited reduced adhesion and invasion to human epithelial cell INT-407 and human enterocyte like epithelial CaCo-2 cells (Kim et al., 2010). Similar observations were also made in avian pathogenic *E. coli*, where inactivation of OmpF and OmpC were shown to significantly hamper its adhesive, invasive and colonization abilities (Hejair et al., 2017). Previous report in clinical *Enterobacter aerogenes* isolates, suggested the significance of OMPs in modulating membrane permeability, which in turn affected its susceptibility to antibiotics and colonization abilities in nematodes (Lavigne et al., 2012).

Overall literature suggests OMPs to play a significant role in conferring antibiotic resistance, boosting virulence properties of many opportunistic bacterial pathogens (Delcour, 2009; Sato et al., 2017) and helping the pathogen to adapt to adverse situations. Despite these observations, population level association studies between OMPs, antibiotic resistance and virulence is still not completely explored (Silva and Mendonça, 2012). This prompted us to question, whether there exists any association between the OMPs, antibiotic resistance and virulence in a given population. Such pilot scale population association studies are important to understand the potential health risks associated with opportunistic multidrug resistant environmental bacteria.

## Materials and Methods

### Bacterial Strains and Growth Conditions

Pure cultures of 20 environmental *Enterobacter* isolates, obtained from aquatic environment from Jamshedpur, India, in a prior study (Table S1) (Singh et al., 2017) and, 22 clinical *Enterobacter* isolates, obtained as pure cultures from wound, pus, and urine samples of patients admitted to tertiary care hospitals in Bhubaneswar, India (Table S1) were included in this study. Isolation of pure colonies of clinical isolates was done at the tertiary care hospitals by using MacConkey and/or CLED agar followed by identification using routine biochemical tests and automated bacterial identification system VITEK2 (bioMerieux, USA) or a Microscan Walkaway 40/96S system. Bacterial pure cultures were maintained in nutrient agar stab culture at room temperature (Himedia, India). Unless otherwise mentioned, all experiments were carried out at 37°C and rotation of 220 per minute in shaker incubator (New Brunswick, USA) and performed in triplicates. Following sub-culture, the isolates were cryopreserved in 20% glycerol at −80°C. *Salmonella typhi*i ATCC 13324 and *E. cloacae* ATCC 13047 were used as positive controls in *in-vitro* cell adhesion and invasion studies. *Escherichia coli* ATCC 25922 was used as control strain for antibiotic disk diffusion test.

### Antibiotic Resistance Profiling

Antibiotic susceptibility profiles of environmental (*n* = 20) and clinical (*n* = 22) *Enterobacter* isolates was determined by disk diffusion method (Bauer et al., 1966) using commercially available antibiotic disks (Hi-Media, India) representing all the major groups *viz*. β-lactams including cephalosporins, aminoglycosides, quinolones, macrolides, polypeptide, sulfonamides and others (Table S2). The diameter of the inhibition zone was recorded after overnight incubation and interpreted following CLSI standards for Enterobacteriaceae (CLSI., 2017).

As described by Krumperman (1983), multiple antibiotic resistance (MAR) index for each isolate was calculated using the following equation:

$$\begin{array}{l} \text{MAR}\text{ index }=a/b \end{array}$$

where, “*a*” represents number of antibiotics to which isolate is resistant and “*b*” represents the number of antibiotics to which isolate was exposed.

### Screening of OMP Genes by Multiplex PCR

Hexaplex PCR was developed to detect the presence of genes encoding prototype porins *viz*. OmpA, OmpF, OmpX, OmpC, LamB, and FhuA, using oligonucleotides designed for this study (Table S3). Briefly, bacterial cell lysates were prepared by heat denaturation followed by snap chilling, and were used as the source of template DNA. Final volume of 100 μl reaction mixture, contained 20 μl of (5X) Gotaq Flexi buffer (Promega, USA), 2 μl of (2.5 mM/dNTP) dNTP mix (Promega, USA), 2.5 μl of 25 mM MgCl2, 1.5 μl of (10 μM) forward, and (10 μM) reverse primer each, 2.5 μl of cell lysate as template DNA and 0.2 μl (100U) of Gotaq flexi DNA polymerase (Promega, USA) in a thermal cycler (Eppendorf, Germany). Reaction cycles was programmed with an initial denaturation at 94°C for 2 min, followed by 35 cycles with initial denaturation at 94°C for 45 s, annealing temperature of 53°C for 45 s, and extension at 72°C for 45 s which was followed by a final extension at 72°C for 10 min. PCR products were run in 1.5% agarose gels prepared in 1X Tris-Acetate Buffer (TAE) and gel images were recorded using the gel documentation system (Bio-Rad, USA). Following gel extraction and purification the nucleotide sequence of PCR products was determined in an automated 3130XL Genetic Analyzer (Applied Biosystems, USA).

### Slot Blot Hybridization for Confirming Presence of OMP Genes

In addition to the PCR and sequence confirmation, we had validated our results by slot blot hybridization utilizing bacterial genomic DNA extracted from clinical and environmental isolates using Gentra Puregene bacteria/yeast DNA isolation kit (Qiagen, USA), as described previously (Singh et al., 2002). Bacterial genomic DNA was extracted using Gentra Puregene bacteria/yeast DNA isolation kit (Qiagen, USA). Two hundred nanogram of genomic DNA were lysed with equal volume of denaturation buffer (0.5 M NaOH, 1.5 M NaCl). Slot blots were prepared with nylon filters (Hybond; Amersham International, London, UK) using PR648 Slot Blot Manifold (GE healthcare Life Sciences, USA) and neutralized in neutralizing solution (0.5 M Tris-HCl [pH 8.0], 1.5 M NaCl). Finally, the liberated DNA was fixed to nylon membranes by exposure to UV light for 1 min (1800 × 100 uJ/cm^2^) in a UV-crosslinker, in accordance with the manufacturer's instructions.

The membrane was probed using purified PCR products (whose identity was confirmed by sequencing) randomly labeled with [α-32P] dCTP (3,000 Ci/mmol, BARC, Bombay, India). The membrane was hybridized at 65°C in phosphate buffer containing 500 mM Na_2_HPO_4_ (pH 7.2), 7% (wt/vol) sodium dodecyl sulfate, 1 mM EDTA, and 1% (wt/vol) bovine serum albumin. Hybridized blots were washed once in 2 × SSC buffer (1 × SSC is 0.15 M NaCl with 0.015 M sodium citrate) for 5 min at room temperature, two times in 2 × SSC-0.1% sodium dodecyl sulfate for 10 min at 65°C, and once in 0.1 × SSC-0.1% sodium dodecyl sulfate for 15 min at 65°C. Autoradiographs were developed from the hybridized filters with the Bio-Rad Phosphor Imager screen and visualized in a Phosphor Imager (Bio-Rad, USA).

### Phenotypic Detection of Virulence Factors

Clinical and environmental MDR *Enterobacter* isolates with MAR index >= 0.3 (taken as cut off) and presence of OMPs, were checked for presence of different virulence factors (Table S4). The presence of type-1 fimbriae in clinical isolates was determined by Hemagglutination assay (Hennequin and Forestier, 2009). The biofilm formation ability of both clinical and environmental *Enterobacter* isolates was also determined (Figure S1) by using crystal violet method (Stepanović et al., 2000), with some modifications reported previously (Singh et al., 2017), and were interpreted as weak, moderate and strong biofilm producer. Bacterial resistance to human serum was assessed following the protocol as described earlier (Sahly et al., 2004), with and without heat-inactivated serum. Serum resistance profile was categorized into grade-1 being non-resistant to grade-6 with highest level of resistance (Table S5), as described previously (Sahly et al., 2004).

### Determination of Host-Dependent Virulence Factors

Essential steps for initiation of pathogenesis include adhesion and invasion to the host cells. Based on our previous observations, six environmental and six clinical MDR *Enterobacter* isolates exhibiting a MAR index >= 0.3, positive for presence of different combinations of OMPs and positive for serum resistance and biofilm production, were further selected for *in-vitro* gentamycin protection assay.

#### In-vitro Adhesion and Invasion in Murine Macrophage

To confirm the adhesive and invasive properties of selected MDR clinical and environmental *Enterobacter* isolates gentamicin protection assay, with *Enterobacter* isolate ATCC 13047 as control, was performed. Briefly, RAW 264.7 murine macrophage cell line was maintained in RPMI-1640 media (Himedia, India) supplemented with antibiotic cocktail containing 1X penicillin-streptomycin and 250 μg of amphotericin (Himedia, India) and 10% FBS (PAN Biotech, India). Initially, RAW 264.7 cells were trypsinized, counted with and plated into 12 well tissue culture plate with 2 × 10^5^ cells/ml in each well without antibiotics and incubated overnight at 37°C with 5% CO_2_ in New Brunswick incubator (Eppendorf, India). Bacterial pure culture was inoculated into 3 ml of tryptic soy broth (Himedia, India) and incubated at 37°C overnight. Culture was then diluted 1:1000 in fresh 5 ml of Tryptic Soy Broth and allowed to grow for 3–4 h till OD_600nm_ reaches to 0.6–0.8. Bacterial cultures were then centrifuged at 4,500 rpm for 10 min at 4°C, bacterial pellet was washed once with in 1X PBS (pH 7.4) and before challenge was mixed with RPMI-1640 without antibiotics. For all further bacterial challenge studies, an optimized multiplicity of infection (MOI) 1:50 and infection time of 60 min at 37°C with 5% CO_2_ was used. Using aforementioned conditions, co-cultured plates following incubation, were washed twice with 1X PBS (pH 7.4). Of them, one plate was further incubated with RPMI-1640 containing 200 μg/ml of gentamycin. After incubation, plates were washed twice with PBS and cells were lysed with 0.05% Triton-X100 and plated onto Tryptic Soya Agar. Plates were incubated overnight at 37°C for enumeration of viable counts. The observations were tabulated and statistical analysis of the data obtained from three individual experiments was performed. Moreover, *in-vitro* cell adhesion and invasion frequency were calculated for individual isolates as mentioned earlier (Wilson et al., 2011) to enumerate the percent fraction of populations infecting host cells.

#### Determination of Reactive Oxygen Species (ROS) Production

On the basis of *in-vitro* cell adhesiveness and invasiveness property, three clinical (EspIMS6, EcTATAH41, ATCC 13047) and three environmental (DL4.3, DL5.1, and SR4.9) MDR *Enterobacter* isolates were selected further. The selection of these isolates was based on the results of gentamycin protection assay, where isolates having higher invasive ability were selected for reactive oxygen species (ROS) production. To assess the production of ROS, we had infected the freshly isolated neutrophils with these six MDR *Enterobacter* isolates. Briefly neutrophils were extracted from peripheral blood from healthy volunteers using histopaque 1119 (Sigma Aldrich, USA) and Percoll (Sigma Aldrich, USA) gradient method, as described previously (Sarkar et al., 2012).

Cells were counted using hemocytometer and 1 × 10^6^ cells were seeded in a 48-well tissue culture plate. Bacterial cells were challenged at MOI 1:50 in triplicate and plates were incubated for 1 h at 37°C with 5% CO_2_. After incubation, infected neutrophils were centrifuged at 2,200 rpm for 10 min at room temperature; pellet was suspended with 1X PBS pH 7.4 (Himedia, India). The cell suspension was transferred to FACS tubes and then incubated with ROS indicator fluorescent dye Dihydrorhodamine 123 (Thermofisher Scientific, USA) at a final concentration of 1 μM at 37°C in a water bath for 5 min. Samples were immediately processed for acquisition and flow cytometric assessment of ROS production by BD FACS Calibur^TM^ flow cytometer (BD Biosciences, USA) and analyzed by CellQuest Pro software (BD Biosciences, USA).

### Whole Genome Sequencing of Two *Enterobacter* Isolates

In a prior work, we have reported draft genome sequence of environmental *Enterobacter cloacae* isolate DL4.3 (showing multi-drug resistance phenotype) and clinical *Enterobacter cloacae* isolate EspIMS6 (having extreme drug resistance phenotype; Mishra et al., 2017). To further understand and validate experimental results obtained, we analyzed our draft genomic sequences in an internet-based platform (Center for genomic epidemiology http://www.genomicepidemiology.org), which provides a platform for rapid analysis of whole genome DNA-sequences and retrieve information from the sequence data.

### Statistical Softwares Used

Bionumeric software (v.7.0, Applied Maths, Biomeriux, USA) was used to analyze antibiotic resistance pattern of the isolates used in the study and dendrogram was generated using UPGMA algorithm inbuilt in the software. Pearson correlation coefficient of OMPs and *in-vitro* adhesion and invasion frequency were generated using XLSTAT software (v. 2017, www.xlstat.com/en/). Analyzed matrix was then plotted in biplot to assess the association between attributes and the pathogenic potential was derived from the biplot generated from principal component analysis. GraphPad prism v. 7.0 was used to generate graphs from *in-vitro* infection assays.

## Results

### Antibiotic Susceptibility Profile of *Enterobacter* Isolates

**Figure 1:**
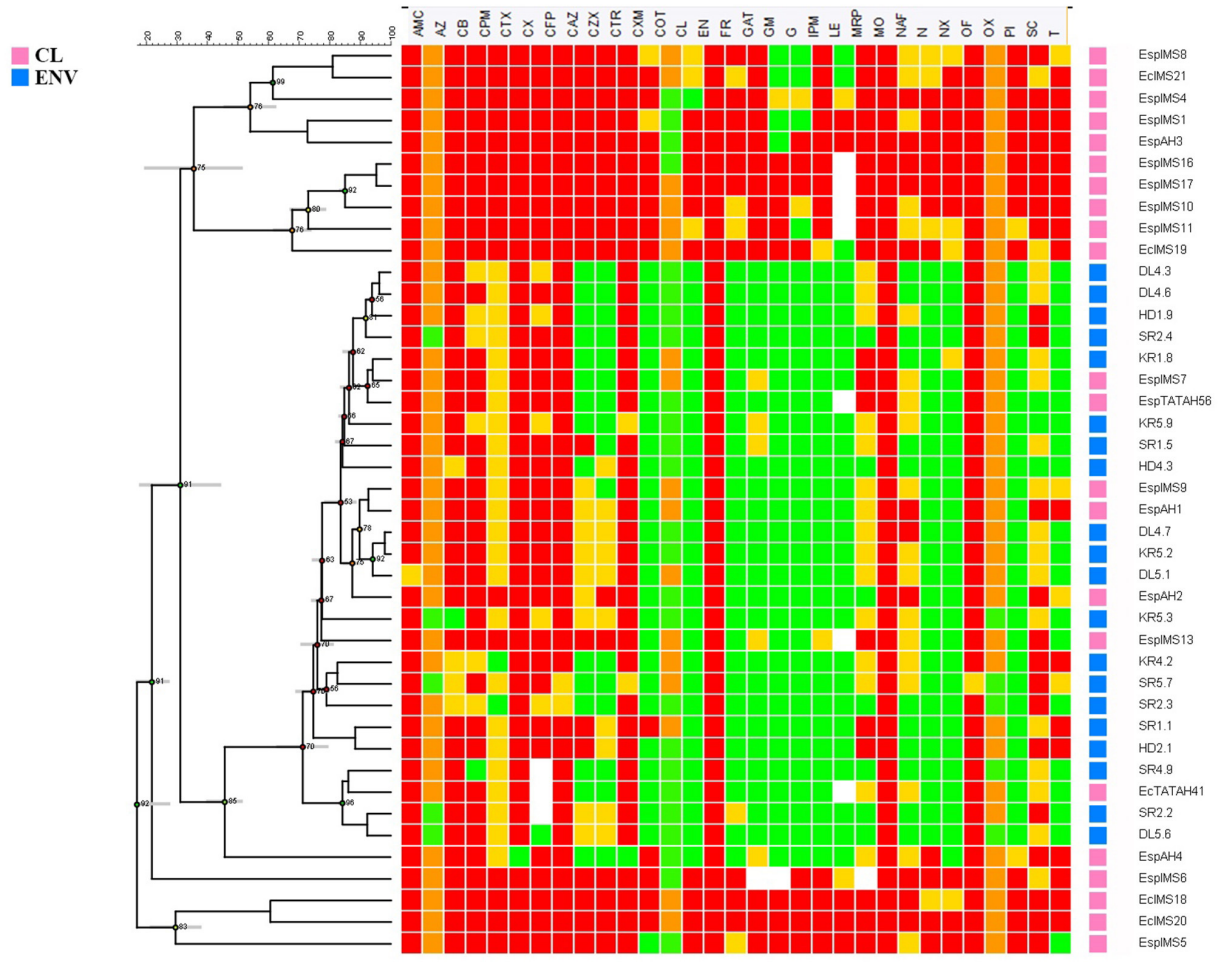
Comparative antibiotic susceptibility profile of environmental and clinical *Enterobacter* isolates. Antibiotic susceptibility of environmental (*n* = 20) and clinical (*n* = 22) *Enterobacter* isolates toward 40 different antibiotics belonging to different classes was performed by disk diffusion method. Zone of inhibition was recorded to represent the Resistant (Red), Intermediate (Yellow) and Susceptible (Green) isolates. The antibiogram profiles of all these isolates are represented here as a heat map with dendrogram, which was generated using Bionumerics v7.0 software.

Antibiogram profile of the isolates was determined against 40 antibiotics representing major classes of therapeutic agents. Results revealed marked differences in antibiotic susceptibility profiles of environmental and clinical isolates used in the study (Figure 1). Alarmingly 50% (*n* = 11) of clinical isolates were resistant to colistin, and 30% (*n* = 7) of them were resistant to imipenem and meropenem, but all environmental isolates were sensitive to the above mentioned drugs (Figure 1). The clinical isolates were completely resistant to β-lactams, first and second generation of cephalosporins while around 75% of clinical isolates were resistant to third generation cephalosporins *viz*. ceftriaxone, cefotaxime, ceftazidime. All of the clinical isolates (*n* = 22) showed resistance toward fourth generation cephalosporins like cefpirome and cefepime (Figure 1). In contrast, environmental isolates were mostly resistant toward first generation of cephalosporins like cefuroxime. While majority of the environmental *Enterobacter* isolates were susceptible to quinolones, resistance toward the drug was exhibited by more than 50% of clinical isolates.

### Multiplex PCR for Simultaneous Detection of OMP Genes

**Figure 2:**
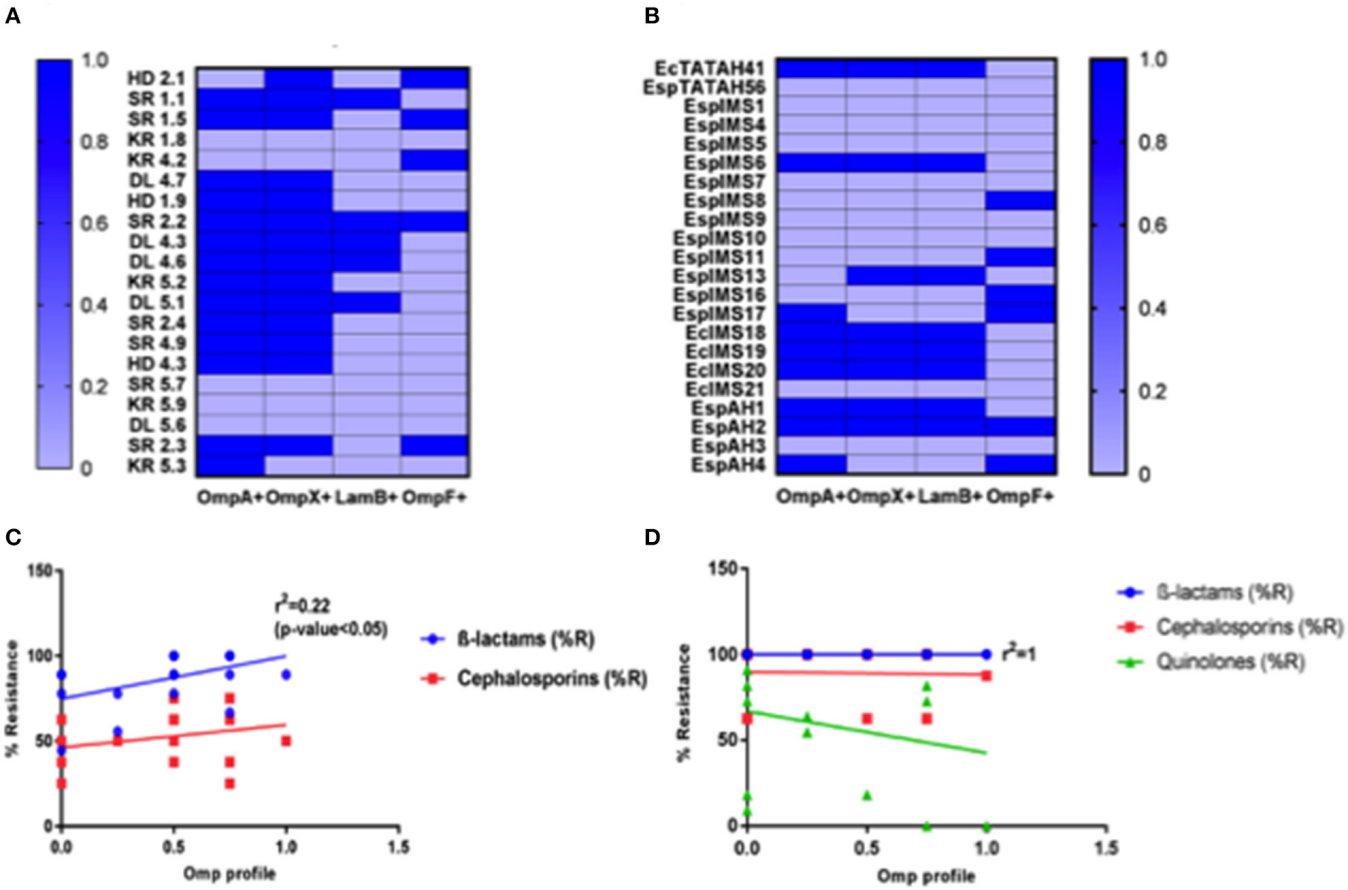
Determination of OMP profile and its association with antibiotic resistance. Distribution of *OmpA, OmpX, LamB*, and *OmpF* in environmental **(A)** and clinical **(B)** *Enterobacter* isolates is presented, as deduced from multiplex PCR and slot blot hybridization experiments. OMP profile of individual isolates was then compared with their respective % resistance toward β-lactams, cephalosporins antibiotics to assess the association of OMPs with observed drug resistance, presented here as linear regression curve for environmental **(C)** and clinical **(D)** *Enterobacter* isolates, done by GraphPad Prism software.

We developed a hexaplex PCR for screening of different OMPs present in *Enterobacter* isolates under study. Hexaplex PCR followed by slot blot hybridization and sequencing of the purified PCR products confirmed the presence of *OmpA, OmpX, LamB*, and *OmpF* in the isolates (Figures 2A,B and Figures S2A,B, S3). Results indicated that majority of environmental isolates (*n* = 13) were positive for *OmpA* and *OmpX* (Figure 2A), out of which five were also positive for *LamB* and, two co-harbored *OmpF*. In contrast, eight clinical isolates co-harbored *OmpA, OmpX*, and *LamB*; out of which two isolates were positive for *OmpF* (Figure 2B).

It was interesting to note that none of the isolates tested were positive for presence of *OmpC* gene (Figures S2A,B). When investigated for the presence of substrate-specific porins such as *LamB* and *FhuA* in the *Enterobacter* isolates, we did not find any isolate positive for *FhuA*. However, *LamB* was present in 25% of environmental and 37% of clinical isolates, making *LamB* as the third most abundant OMPs, among *Enterobacter* isolates (Figures 2A,B).

### Association of OMPs With Antibiotic Resistance

We analyzed association between OMPs and antibiotic resistance in the sample population under study using linear regression. Among the environmental isolates, we observed an association between OMP positive isolates and β-lactam, cephalosporins resistance (Figure 2C). Moreover, environmental isolates possessing *OmpA* and *OmpX* (*n* = 11) exhibited MDR as against those not harboring *OmpA* and *OmpX genes* (results not shown). It also indicated that isolates devoid of *LamB* and *OmpF* were resistant toward higher number of β-lactam, cephalosporins antibiotics. On the contrary, it was well-understood that OMPs in clinical *Enterobacter* isolates had significantly less or no association with observed antibiotic resistance (Figure 2D), attributed to presence of multiple chromosomally encoded resistance determinants.

### Phenotypic Detection of Virulence Factors

Clinical isolates (*n* = 7) and environmental *Enterobacter* isolates (*n* = 5) along with prototype strain, *Enterobacter* ATCC 13047, exhibiting MAR index >= 0.3 and positive for presence OMPs, were checked for presence of virulence factors such as type-1 fimbraie, biofilm formation and serum resistance (Table S4). Hemagglutination assay for detection of type-1 fimbriae revealed that all environmental and clinical *Enterobacter* isolates tested were positive for fimbriae. Environmental isolates DL4.3, DL4.6, SR5.7, and DL5.1 displayed low and moderate serum resistance, whereas SR4.9 showed high serum resistant phenotype. On the contrary, clinical isolates EcTATAH41, EspIMS6, and EcIMS18 were highly serum resistant indicating strong pathogenic potential. Most of the clinical *Enterobacter* isolates tested, including EcTATAH41, EspIMS6, EcIMS21, EspAH3, and EspAH4 were strong biofilm producer unlike environmental *Enterobacter* isolates like SR4.9, DL4.3, and DL4.6 that were weakly adherent in nature.

### *In-vitro* Cell Adhesion/Invasion of MDR *Enterobacter* Isolates

Bacterial challenge to murine macrophage RAW 264.7 cell line evaluated the pathogenicity of *Enterobacter* isolates. Compared to *in-vitro* cell-attachment and invasion potential of control pathogenic strain of *Salmonella typhii* isolate ATCC 13324, *Enterobacter* isolates used in the present study were categorized into three major groups: (A) Highest pathogenic potential [P.P.(*Entero*.) ≥ P.P.(*S. typhii*)]; (B) Moderate pathogenic potential [P.P.(*Entero*.) ≤ P.P.(*S. typhii*)] and (C) Minimal pathogenic potential [P.P.(*Entero*.) < < P.P.(*S. typhii*)]; where P.P. refers to pathogenic potential of the tested organism.

**Figure 3:**
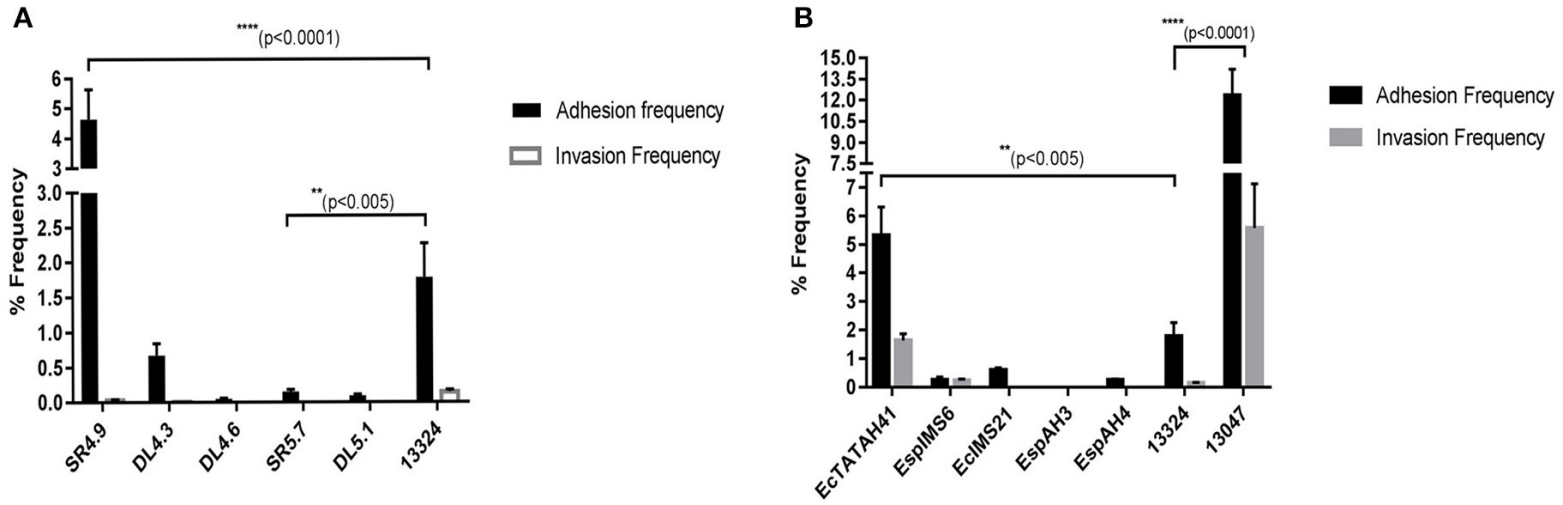
Cell adhesion and invasion frequency of *Enterobacter* isolates. The bar graph represented the % adhesion and invasion frequency of selected MDR environmental **(A)** and clinical **(B)** *Enterobacter* isolates, which was calculated by the % ratio of the number of viable bacterial cells (as in mean log_10_CFU/ml) after infection to RAW 264.7 cell line and the initial inoculum given. Statistical significance was determined using two-way ANNOVA test using GraphPad Prism software and *S. typhii* ATCC 13324 as control, where ****p < 0.0001 and **p < 0.005.

Cell attachment assay revealed that environmental isolate SR4.9, with 4.65% of adhesion frequency showed highest *in-vitro* cell attachment, emulating results as observed with *Salmonellae typhii* ATCC 13324 having adhesion frequency of 1.78% (Figure 3A). Further, with ~0.1% adhesion frequency environmental isolates DL5.1 and SR5.7 exhibited moderate attachment (Figure 3A). However, none of the environmental isolates could show cell invasion ability *in-vitro* (Figure 3A).

On the contrary, clinical *Enterobacter cloacae* ATCC 13047 isolate with 12.34% adhesion frequency and 5.57% invasion frequency, displayed highest cell attachment and cell invasion *in vitro*, respectively (Figure 3B). Moreover, clinical isolate EcTATAH41, EspIMS6, and EcIMS21 showed moderate cell attachment with the former also exhibiting moderate cell invasion *in vitro* (Figure 3B). It was worth noting that, the clinical isolate EspIMS6 exhibited almost complete invasion following attachment *in vitro* (Figure 3B), as evident from their nearly similar adhesion and invasion frequency. With the % ratio of invasion to adhesion frequency, it was noted that clinical isolates EcTATAH41, EspAH3, and Ec13047 showed 30–45% of invading populations while EspIMS6 displayed >90% of invading populations to macrophage cells.

### Flow Cytometric Assessment of ROS Production

**Figure 4:**
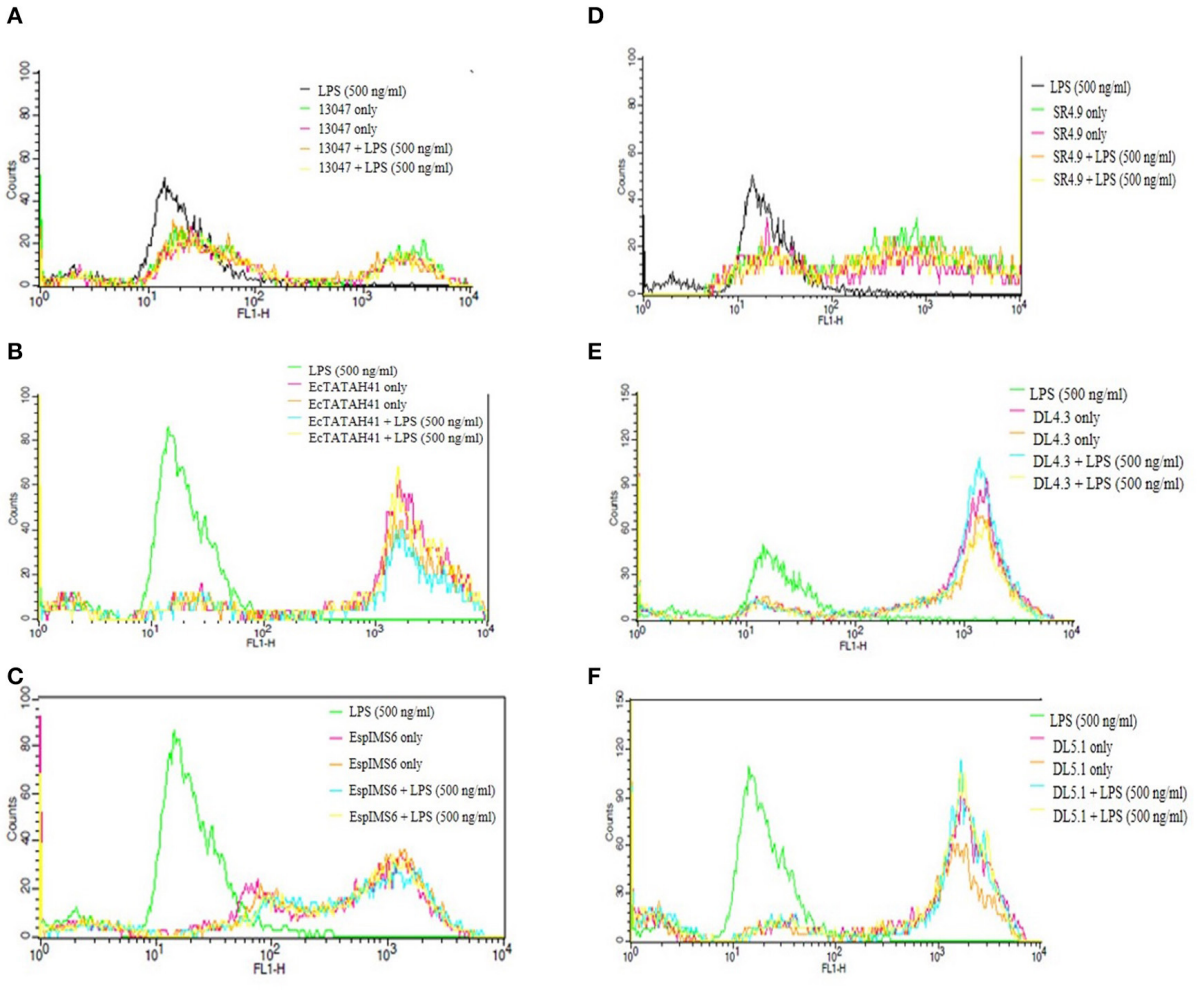
Determination of ROS generation in neutrophils challenged with MDR *Enterobacter* isolates. The histograms of Dihydrorhodamine 123 fluorescence emitted due to activation of neutrophils by the MDR pathogens with and without LPS (in duplicate) and LPS control were recorded and represented here with gated populations for clinical *E. cloacae* ATCC 13047 **(A)**, EcTATAH41 **(B)** and EspIMS6 **(C)** and environmental SR4.9 **(D)**, DL4.3 **(E)**, DL5.1 **(F)** *Enterobacter* isolates.

Production of reactive oxygen species by neutrophils plays a pivotal role in innate immune response to pathogens. Hence, based on the results of gentamycin protection assay, we determined the activation of neutrophils by estimating the ROS production upon co-culture of neutrophils with six MDR *Enterobacter* isolates. Flow cytometry results of ROS generation by *Enterobacter* isolates as against LPS control were compiled (Figures 4A–F). Results of this study suggested that clinical MDR isolates EcTATAH41 (Figure 4B) and EspIMS6 (Figure 4C) produced significant amount of ROS, which was evident from the shift in fluorescence indicating activation of neutrophils upon infection. It was interesting to note that environmental *Enteorbacter* isolates DL4.3 (Figure 4E) and DL5.1 (Figure 4F) too were capable of producing ROS similar to clinical counterparts. On the other hand, aquatic isolate SR4.9 (Figure 4D) and ATCC strain 13047 (Figure 4A) displayed minimal production of ROS upon neutrophil infections.

### Association of OMPs With Pathogenic Potential of *Enterobacter* Isolates

After determining the presence of different virulence factors, we analyzed the association of OMPs with *in-vitro* cell adhesion and invasion frequencies. From the Pearson correlation matrix, it was evident that *OmpA* and *OmpX* were strongly correlated (*r* = 0.8). *LamB* displayed a positive correlation with other OMPs; significantly with *OmpX* (*r* = 0.8) followed by *OmpA* (*r* = 0.6) (Table S6). Further, a positive correlation between *OmpF* and invasion frequency (*r* = 0.6) was observed.

**Figure 5:**
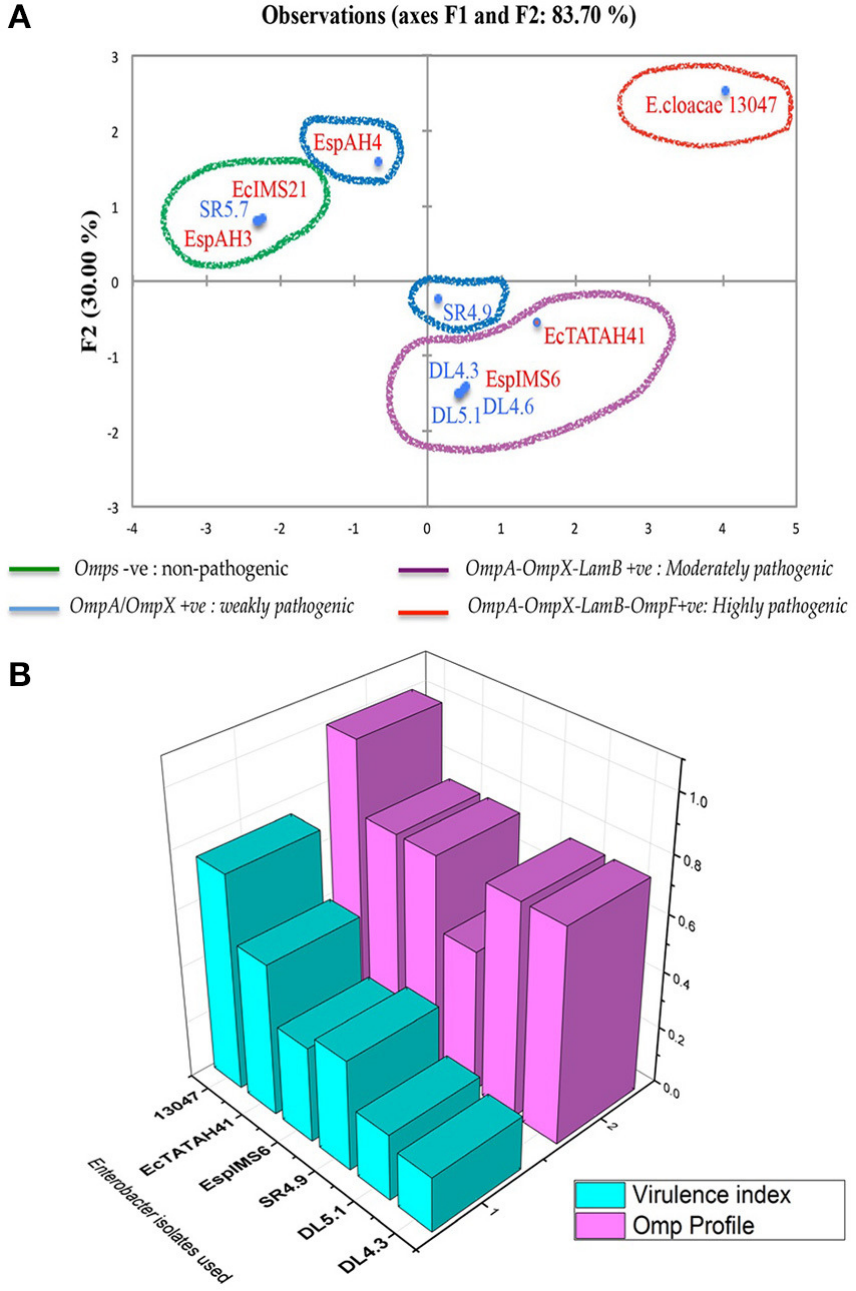
Association of OMPs with pathogenic potential in clinical and environmental *Enterobacter* isolates. Principal component analysis represented as biplot **(A)** associating pathogenic potential and expression of OMPs has categorized into four groups, where organisms in red indicated clinical *Enterobacter* isolates and in blue indicated environmental *Enterobacter* isolates. **(B)** is a 3D plot showing the association between of OMP profile with virulence index of individual *Enterobacter* isolates.

The results of this Pearson correlation matrix were further evaluated, where the *Enterobacter* isolates were grouped into four quadrants based on their OMP profile and respective *in–vitro* adhesion and invasion frequencies (Figure 5A). *E. cloacae* ATCC 13047 that was positive for *OmpA, OmpX, LamB, OmpF* exhibited highest adhesion-invasion frequency, indicating its greater pathogenic ability. Further, clinical (EcTATAH41 and EspIMS6) and environmental (DL4.3, DL4.6, DL5.1) isolates positive for *OmpA, OmpX*, and *LamB* showed moderate and weak adhesion-invasion frequency, respectively (Figure 5A). In addition, environmental isolate SR4.9 that was positive for *OmpA* and *OmpX* showed strong cell-adhesive property. Interestingly, EspAH4 that was positive only for *OmpA* displayed weaker cell adhesive quality, whereas isolates like SR5.7, EcIMS21, EspAH3 which were devoid of OMPs, did not show *in-vitro* adhesion or invasion ability (Figure 5A).

To give a clear picture of this observed association of OMPs with pathogenic potential, we calculated the virulence index of each of these six MDR isolates taking into considerations: presence of fimbriae, serum resistance, biofilm production, adhesion and invasion frequency (using the formula virulence index = total no. of virulence factors possessed/total no. of virulence factors tested), and analyzed association of virulence index with their respective OMP profile (Figure 5B). This data clearly showed that clinical isolates (e.g., EcTATH41, EspIMS6, and ATCC 13047) exhibited greater pathogenic ability as observed from their higher virulence index and OMP profile. Nonetheless, environmental isolates (including SR4.9, DL5.1, and DL4.3), though harbored multiple OMPs, they displayed minimal pathogenic ability, that could be attributed to lower virulence indices.

### Virulence Mechanisms of *E. cloacae* Isolates Revealed by Genome Analysis

**Figure 6:**
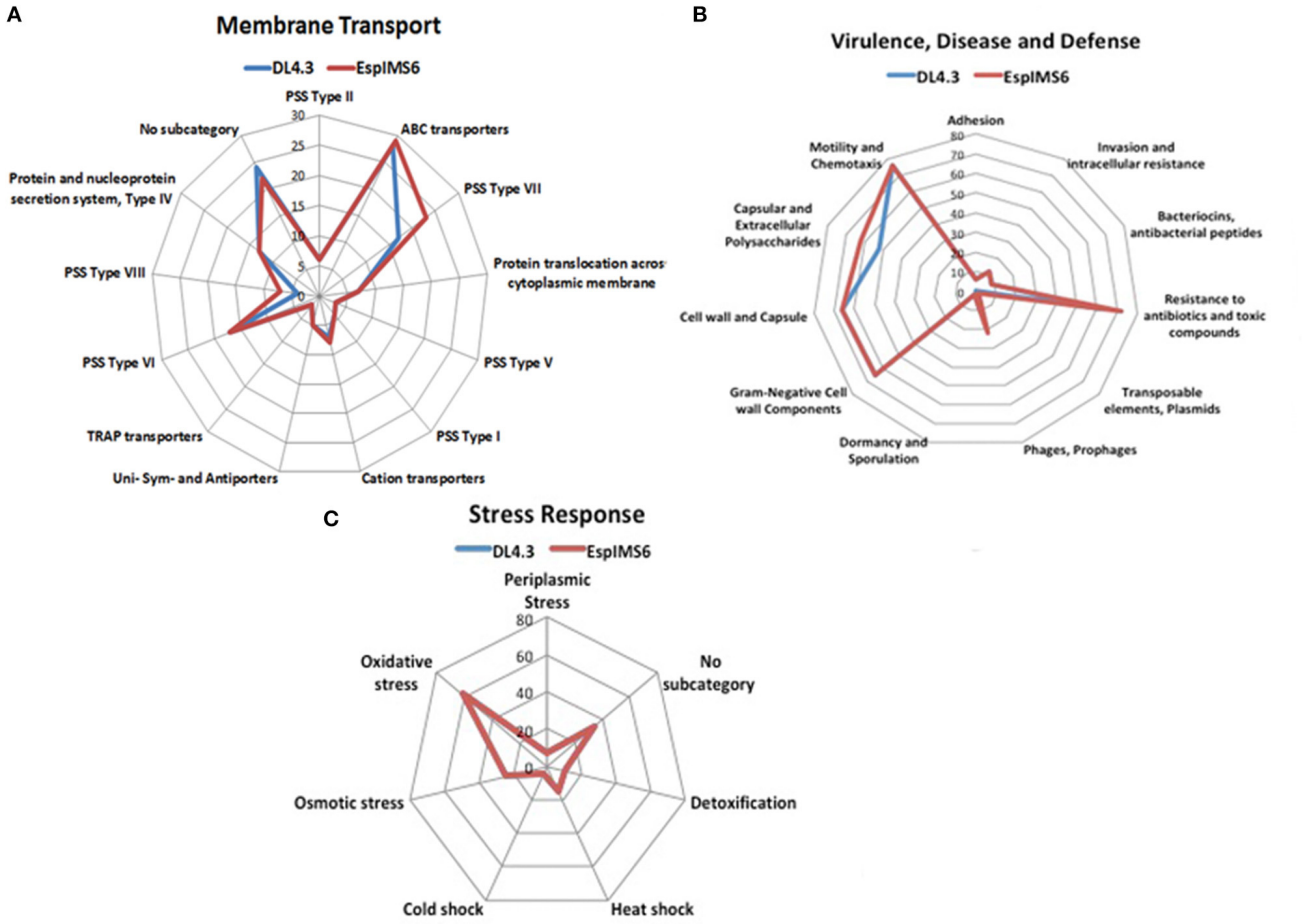
Genetic makeup of environmental (DL4.3) and clinical (EspIMS6) *Enterobacter* isolates. Spider plots generated using the number of genes involved in each function revealed critical genomic differences and similarities between wild type environmental DL4.3 and clinical EspIMS6 isolate in different categories like **(A)** membrane transport, **(B)** virulence, disease and defense, and **(C)** stress response.

In a prior work, we had reported draft genome sequence of DL4.3 (environmental-aquatic isolate) and EspIMS6 (Clinical-UTI isolate; Mishra et al., 2017). To further understand and validate experimental results obtained, we analyzed sequence data further. In both *E. cloacae* isolates, number of genes associated with functions were in the order carbohydrates > amino acids and derivatives > protein metabolism > cofactors, vitamins, prosthetic groups, pigments > RNA metabolism > cell wall and capsule > membrane transport (Figure S4). We had also segregated individual category and its sub-systems involved to get a blueprint of the similarities and dissimilarities amongst these two *E. cloacae* isolates (Figures 6A–C).

In case of membrane transport machinery, both isolates harbored ABC transporters, cation transporters along with protein secretion systems. However, protein secretion system type VII and type VIII were more prevalent in clinical isolate EspIMS6 (Figure 6A). Both the organisms were genetically similar in terms of their stress response ability, including oxidative stress, osmotic stress, heat shock, cold shock etc. (Figure 6C). Moreover, in case of virulence, defense and disease subcategory (Figure 6B), both DL4.3 and EspIMS6 contained machineries for flagellar motility, chemotaxis, capsules, antibacterial peptides, invasion and intracellular resistance. But, clinical isolate EspIMS6 outnumbered DL4.3, in terms of capsular and extracellular polysaccharides, resistance to antibiotics and toxic compounds, phages and prophages (Figure 6B). Interestingly, clinical isolate EspIMS6 also possessed transposable elements and plasmids, which were absent in the environmental isolate DL4.3 (Figure 6B).

## Discussion

In the present study, we utilized an integrated approach investigating antibiotic resistance profile, presence of outer membrane proteins, virulence factors and utilizing genome sequence data to understand pathogenic potential and extent of threat posed due to multidrug resistant environmental *Enterobacter* isolates. In a previous study conducted by us (Singh et al., 2017), we had quantitatively evaluated threat posed by multidrug-resistant bacteria from environment both at population and genus level. Also, we had reported environmental multidrug *Enterobacter* isolates posed no threat. Detailed investigation in the present study corroborated well with our previous observations.The environmental isolates exhibited low ROS production in neutrophils, lacked OmpC and lacked cell invasion abilities. In contrast clinical isolates produced higher amounts of ROS in neutrophils, possessed all OMPs screened for, and exhibited extremely high invasive and adhesive capabilities. Whole genome sequencing revealed presence of integrons, type VII and type VIII secretion systems in clinical isolate only. Thus, findings of our studies indicate that multidrug resistant environmental *Enterobacter* isolates investigated in this study are limited in their pathogenic ability compared to clinical isolates.

We investigated environmental (*n* = 20) and clinical (*n* = 22) *Enterobacter* isolates, belonging to two major species- *E. cloacae* and *E. aerogenes*, indicating ubiquitous existence of these two species in aquatic and clinical environments. As reported by us previously (Singh et al., 2017), majority of the environmental *Enterobacter* isolates were multi-drug resistant (*n* = 15), whereas, majority of the clinical isolates tested in this study, exhibited resistance to all the antibiotics tested and accordingly could be categorized as MDR (*n* = 13), extreme drug resistant (XDR) (*n* = 6) and pan drug resistant (PDR) (*n* = 3) (Paterson and Doi, 2007). These results indicated the emergence of potential multi-drug resistant strains of *Enterobacter* in both aquatic environment and clinical settings.

Previous reports have cited existence of enzymatic barrier coupled with porin loss/reduction in *Enterobacter* spp. to play a significant role in eliciting resistance toward carbapenems like ertapenem and/or imipenem (Doumith et al., 2009; Lavigne et al., 2012; Yang et al., 2012). Study conducted on the dynamic changes in membrane permeability of *E. aerogenes* clinical isolates subjected to imipenem treatment, revealed no difference in expression of OmpA and OmpX between resistant and intermediate susceptible isolates (Lavigne et al., 2012). Majewski et al. (2016) have studied OMP expression in carbapenem-resistant *Enterobacter* isolates. The authors have reported either downregulation of OmpF and OmpC gene and/or OmpC-directed polarization of the outer membrane to affect carbapenem resistance. This altered outer membrane protein balance in the context of OmpF/OmpC greatly regulates the β-lactam resistance by selecting porins with preferable transmembrane channel diameter (Yigit et al., 2002). This prompted us to investigate the distribution of prototype OMPs (both non-specific and substrate-specific porins) in clinical and environmental *Enterobacter* isolates.

Hexaplex PCR revealed predominance of *OmpA* and *OmpX* in the isolates under study, coinciding with earlier reports, which suggest these two OMPs to be integral part of gram-negative bacterial membrane (Dupont et al., 2004). Overexpression of *OmpX* in *E. coli* and *E. aerogenes* strains was found to reduce expression of non-specific porins i.e., *OmpC* and *OmpF*, leading to restricted permeability of β-lactams (Dupont et al., 2004; Viveiros et al., 2007). A study conducted by Jaskulski et al. (2013) with carbapenem resistant *E. cloacae* isolates (*n* = 106), reported expression of OmpF and OmpC protein in only 6.6% isolates (*n* = 7); out of these seven isolates, only four co-expressed OmpF and OmpC protein. It was interesting to note that none of the isolates we tested, were positive for OmpC. Reduced or no expression of two major non-specific porins, OmpC and OmpF in *E. cloacae* isolates, could be due to point mutations affecting their transcription/translation/insertion into outer membrane (Doumith et al., 2009). We also observed that in our present study *LamB* as the third most abundant OMP, after *OmpA* and *OmpX*. Constitutive expression of LamB was previously reported in many clinical isolates of *Enterobacter aerogenes*, and overproduction of LamB and OmpX was associated with major porin loss (Gayet et al., 2003). Utilizing multiplex PCR based screening, results of our study established differences in occurrence of OMPs in *Enterobacter* spp. isolated from clinical and environmental origin.

Antimicrobial susceptibility profile of the *Enterobacter* isolates had exhibited wide spread resistance to β-lactams and cephalosporins. Hence, we investigated the association of OMPs (*OmpA, OmpX, LamB*, and *OmpF*) in mediating antibiotic resistance. We observed significant association of *OmpA* and *OmpX* with β-lactam and cephalosporin resistance. This indicated probable role of these two porins in mediating resistance to β-lactams in environmental *Enterobacter* isolates. Co-ordinated and similar association of both these OMPs might be, because of the common global regulatory pathways involved in such porin regulation, such as CpxAR and EnvZ/OmpR in response to antibiotics (Dam et al., 2018). OmpA is a multifaceted porin, and is widely conserved in many pathogens like *E. coli, Enterobacter* spp., *Klebsiella* spp., *Acinetobacter baumanii* (Confer and Ayalew, 2013). Apart from maintaining cellular integrity, OmpA plays a vital role in biofilm formation and adherence to biotic and abiotic surfaces (Gaddy et al., 2009). We observed that the clinical isolates which possessed *OmpA* namely, *Enterobacter cloacae* 13047, EspAH4, EcTATAH41, and EspIMS6, to be strong biofilm producers. OmpX, a structural homolog of OmpA has been reported, to be important for bacterial pathogenesis (Maisnier-Patin et al., 2003). This finding corroborates with the recent reports, suggesting that bacterial OMPs play a major role in developing antibiotic resistance, since these porins are responsible for intrusion of antibiotics (Ghai and Ghai, 2018).

We observed higher percent of antibiotic resistant isolates to be negative for *LamB* and *OmpF*, suggesting significant low association of *LamB* and *OmpF* in antibiotic resistance amongst environmental isolates. Though we have discussed loss/reduction of OmpF expression in resistant isolates above, but association of LamB with drug resistance is less explored. We could not find any reports on association of LamB with virulence factors either. But there is one report that explored role of LamB as a vaccine candidate among *Vibrio* species (Lun et al., 2014). LamB porin, known to be responsible for transport of maltose and maltodextrin in gram-negative bacteria, are reported to exhibit poor immunological characteristics. Nonetheless, LamB-one of the first OMPs characterized, are evolutionary significant irrespective of their contribution toward antibiotic resistance and virulence (Koebnik et al., 2000).

Bacterial virulence factors enable the pathogen to replicate and disseminate within host cells in part by evading the host-defense system, hence determination of such virulence factors is important to assess their pathogenic potential (Schroeder et al., 2017). For any opportunistic pathogen like *Enterobacter* spp., cell adherence and invasion are essential steps for successful colonization and subsequent infection. Hence, we investigated the ability of selected MDR aquatic and clinical isolates to adhere and invade murine macrophage cells (RAW 264.7). The prototype *E. cloacae* isolate ATCC 13047 exhibited highest pathogenic potential, as it mimicked adhesive and invasive competence similar to the positive control *S. typhii* ATCC 13324 used in the present study. This was coinciding with an earlier report (Pati et al., 2018), which suggested the *E. cloacae* 13047 to be the most virulent strain of *Enterobacter* spp. known similar to the pathogenic *S. typhii*. Unlike clinical isolates, which displayed moderate cell invasion, none of the environmental isolates showed cell-invasiveness. In addition, greater percentage of invading populations in clinical isolates (EspIMS6, EcTATAH41, and Ec13047) than aquatic isolates (DL4.3, DL5.1, SR4.9), suggested their higher pathogenic potential. Moreover, upon EspIMS6 infection to macrophage cells, ~90% of adherent cells were actually invading macrophage cells, indicative of its greater pathogenic index. Clinical *Enterobacter* isolates possessed edge over environmental isolates in terms of their biofilm formation, serum resistance and ROS production in neutrophils. Noteworthy was the relatively low ROS production in clinical *Enterobacter* isolates (EspIMS6 and EcTATAH41) as compared to aquatic isolates (DL4.3 and DL5.1), which produced higher ROS in neutrophils. This observation coincided with the reports suggesting that potential opportunistic pathogens reduce ROS level, facilitating their survival and colonization in their target host cells (Spooner and Yilmaz, 2011; Hirschfeld et al., 2017).

Based on the results obtained, we further analyzed the association of OMPs and virulence attributes in MDR *Enterobacter* isolates by principal component analysis. It was evident from the analysis that isolates positive for *OmpA, OmpX, OmpF*, and *LamB* exhibited greater pathogenic ability *in-vitro*, and presence of *OmpF* was found to be associated with higher pathogenic ability. Overall, the findings suggested that presence of OmpF facilitates *Enterobacter* spp. in establishing infection in host cells. Importance of OmpF in adhesive and invasive abilities of avian pathogenic *E. coli* had been currently elucidated (Hejair et al., 2017), which is coinciding with our observations in *Enterobacter* isolates. Significant association between OmpA and OmpX too was evident from the matrix. Similar synergy amongst OmpA and OmpX was reported earlier in *Cronobacter sakazakii* in the context of their invasiveness (Kim et al., 2010). When the virulence index of an organism was determined taking into consideration of all these virulence factors (as mentioned previously), we noticed that the clinical *Enterobacter* isolates exhibited higher virulence index as compared to aquatic isolates, even though they did not have a distinct difference in their respective OMP profile.

The draft genome sequences obtained from aquatic isolate DL4.3 and clinical isolate EspIMS6 validated our earlier observations, as EspIMS6 harbored multiple antibiotic resistance determinants that were reflected in their XDR phenotype. Also, EspIMS6 contained integrons and type VII and type VIII protein secretion systems, making this clinical strain more robust virulent pathogen, even greater than *E. cloacae* 13047, which do possess secretion system type VII but not type VIII (Liu et al., 2013). Presence of genes for bacterial persistence and intracellular survival in clinical EspIMS6 explained its higher pathogenic potential as compared to the aquatic isolate DL4.3. Therefore, such comparative genome analysis helped us to understand the internal genetic background of isolates that is reflected in their observed phenotype (Mishra et al., 2017).

## Conclusion

Put together, the present study indicated association of OMPs with both antibiotic resistance and virulence factors in *Enterobacter* spp in the isolates studied. It was also interesting to note that though environmental *Enterobacter* isolates showed multidrug resistance but possessed limited pathogenic potential, whereas clinical MDR *Enterobacter* isolates possess higher pathogenic index indicative of their potential human health risks. Thus, findings of the present study are significant as it highlights limited fitness of multidrug resistant environmental *Enterobacter* isolates. Such investigations provide much needed information on the pathogenic potential of environmental multidrug resistant bacteria thereby assisting identification of potential high-risk pathogenic populations/clones among opportunistic pathogens like *Enterobacter* spp.

## Data Availability Statement

All datasets generated for this study are included in the article/Supplementary Material.

## Ethics Statement

The present study did not involve human subjects. Approval was obtained from Institutional Biosafety Committee to work on BSL2 organisms. Institutional Ethical Committee approval was obtained for the studies on Human or Animal Cells studies.

## Author Contributions

HM supervised, conceptualized, and designed the study. MM performed the experiments and analyzed the results. SP and DS supported in conducting hybridization experiments and gave critical inputs for preparing the manuscript. SB and AS helped with their expertise in neutrophil-based experiments. MM drafted and edited the manuscript. HM reviewed and finalized the draft. All authors approve and gave their inputs in the final manuscript.

### Conflict of Interest

The authors declare that the research was conducted in the absence of any commercial or financial relationships that could be construed as a potential conflict of interest.
