# Supplementary material for: Antibiotic Resistance Profile, Outer Membrane Proteins, Virulence Factors and Genome Sequence Analysis Reveal Clinical Isolates of Enterobacter Are Potential Pathogens Compared to Environmental Isolates
Source: Front Cell Infect Microbiol. 2020 Feb 21;10:54. doi: 10.3389/fcimb.2020.00054 (PMC7047878; doi:10.3389/fcimb.2020.00054)
Supplement: Supplementary file 1 [file Data_Sheet_1.pdf]

Supplementary material

Table S1A: Details of environmental *Enterobacter* isolates used in this study.

Strain ID	Source of isolation	Genbank accession no.	Lysine	Arginine	Ornithine	Identification
SR 1.1	Subarnarekha River	JQ912523	-Ve	+Ve	+Ve	*E. cloacae*
SR 1.5	Subarnarekha River	JQ912528	-Ve	+Ve	+Ve	*E. cloacae*
KR 1.8	Kharkhai River	JQ912527	+Ve	-Ve	+Ve	*E. aerogenes*
HD 1.9	Hudko Dam	JQ912525	-Ve	-Ve	+Ve	*Enterobacter* spp.
SR 2.2	Subarnarekha River	JQ912529	-Ve	+Ve	+Ve	*E. cloacae*
SR 2.3	Subarnarekha River	JQ912531	-Ve	+Ve	+Ve	*E. cloacae*
SR 2.4	Subarnarekha River	JQ912530	-Ve	+Ve	+Ve	*E. cloacae*
HD 2.1	Hudko Dam	Not deposited	-Ve	+Ve	+Ve	*E. cloacae*
SR 4.9	Subarnarekha River	Not deposited	-Ve	+Ve	+Ve	*E. cloacae*
KR 4.2	Kharkhai River	JQ912520	-Ve	-Ve	-Ve	*Enterobacter* spp.
DL 4.3	Dimna Lake	JQ912514	-Ve	+Ve	+Ve	*E. cloacae*
DL 4.6	Dimna Lake	JQ912515	-Ve	+Ve	+Ve	*E. cloacae*
DL 4.7	Dimna Lake	JQ912516	-Ve	+Ve	+Ve	*E. cloacae*
HD 4.3	Hudko Dam	JQ912519	-Ve	+Ve	+Ve	*E. cloacae*
SR 5.7	Subarnarekha River	JQ912524	-Ve	-Ve	+Ve	*Enterobacter* spp.
KR 5.2	Kharkhai River	JQ912526	-Ve	-Ve	+Ve	*Enterobacter* spp.
KR 5.3	Kharkhai River	JQ912521	-Ve	+Ve	+Ve	*E. cloacae*
KR 5.9	Kharkhai River	JQ912522	-Ve	+Ve	+Ve	*E. cloacae*
DL 5.1	Dimna Lake	JQ912517	-Ve	+Ve	+Ve	*E. cloacae*
DL 5.6	Dimna Lake	JQ912518	-Ve	-Ve	-Ve	*Enterobacter* spp.

Table S1B: Details of clinical *Enterobacter* isolates used in this study.

		Aminoacid decarboxylation			
**Strain ID**	**Specimen sample**	**Lysine**	**Arginine**	**Ornithine**	**Identification**
E Sp. IMS 1	Pus	+Ve	-Ve	+Ve	*E. aerogenes*
E Sp. IMS 4	Tracheal aspirate	+Ve	-Ve	-Ve	*Enterobacter* spp.
E Sp. IMS 5	Wound Swab	+Ve	-Ve	-Ve	*Enterobacter* spp.
E Sp. IMS 6	Urine	-Ve	+Ve	+Ve	*E. cloacae*
E Sp. IMS 7	Urine	-Ve	+Ve	+Ve	*E. cloacae*
E Sp. IMS 8	Urine	+Ve	-Ve	-Ve	*Enterobacter* spp.
E Sp. IMS 9	Pus	+Ve	-Ve	-Ve	*Enterobacter* spp.
E Sp. IMS 10	Pus	+Ve	-Ve	-Ve	*Enterobacter* spp.
E Sp. IMS 11	Unknown	+Ve	-Ve	-Ve	*Enterobacter* spp.
E Sp. IMS 13	Pus	-Ve	+Ve	+Ve	*E. cloacae*
E Sp. IMS 16	Wound Swab	+Ve	+Ve	-Ve	*Enterobacter* spp.
E Sp. IMS 17	Urine	+Ve	-Ve	-Ve	*Enterobacter* spp.
EC IMS 18	Blood	-Ve	+Ve	+Ve	*E. cloacae*
EC IMS 19	Pus	-Ve	+Ve	+Ve	*E. cloacae* sp. *cloacae*
EC IMS 20	Blood	-Ve	+Ve	+Ve	*E. cloacae* sp. *dissolvens*
EC IMS 21	Tracheal aspirate	+Ve	-Ve	-Ve	*E. cloacae*
Esp.TATAH 56	Urine	+Ve	-Ve	-Ve	*Enterobacter* spp.
EC TATAH 41	Urine	-Ve	+Ve	+Ve	*E. cloacae*
E Sp. AH1	Pus	-Ve	+Ve	+Ve	*E. cloacae*
E Sp. AH2	Unknown	+Ve	+Ve	+Ve	*Enterobacter* spp.
E Sp. AH3	Wound swab	+Ve	-Ve	-Ve	*Enterobacter* spp.
EC AH4	Wound swab	-Ve	+Ve	+Ve	*E. cloacae*

Note: '+ve'-Positive, '-ve'-Negative. Lysine, Arginine and Ornithine represented aminoacid decarboxylation pattern.

Table S2: List of antibiotics discs (Himedia, India) used in antibiotic susceptibility assay belonging to different classes are listed, along with their abbreviation and potency of the drug.

Name of the antibiotic	Abbreviation	Potency of the disk (ug)	Name of the antibiotic	Abbreviation	Potency of the disk (ug)
**β-lactams**			**Quinolones**		
Ampicillin-A	**A**	10ug	Nalidixic Acid	**NA**	30
Penicillin-G	**P**	10	Cinoxacin	**CIN**	100
Cloxacillin	**CX**	10	Enoxacin	**EN**	10
Naficillin	**NAF**	1	Ciprofloxacin	**CIP**	5
Oxacillin	**OX**	10	Norfloxacin	**NX**	10
Azlocillin	**AZ**	75	Ofloxacin	**OF**	5
Mezlocillin	**MZ**	75	Pefloxacin	**PF**	5
Piperacillin	**PI**	100	Lemofloxacin	**LO**	10
Ticarcillin	**TI**	75	Levofloxacin	**LE**	5
Carbenicillin	**CB**	100	Sparfloxacin	**SC**	5
Amoxiclav	**AMC**	30	Gemifloxacin	**GM**	5
Cephalothin	**CEP**	30	Gatifloxacin	**GAT**	5
Cefoxitin	**CX**	30	Moxifloxacin	**MO**	5
Cefuroxime	**CXM**	30	**Aminoglycosides**		
Ceftazidime	**CAZ**	30	Gentamycin	**G**	120
Cefotaxime	**CTX**	30	Neomycin	**N**	30
Ceftizoxime	**CZX**	30	Co-Trimaxazole	**CO**	25
Ceftriaxone	**CTR**	30	Azithromycin	**AZM**	5
Cefpirome	**CFP**	30	Chloramphenicol	**C**	30
Cefepime	**CPM**	30	Tetracycline	**T**	30
**Carbapenems**			**Others**		
Meropenem	**MER**	10	Sulphafurazole	**SF**	300
Imipenem	**IMP**	10	Trimethoprim	**TR**	5
**Polypeptides**					
Polymyxin-B	**PB**	300			
Colistin	**CL**	50			

Table S3: List of oligonucleotides used in this study for multiplex PCR of OMPs.

Genes	Primer	Primer sequence (5′-->3′)	Primer Length	Amplicon size (in bp)/Source
OmpF	OmpF-F2	GATCTGTACGGGAAAGCAGTT	21	1001/This study
	OmpF-R2	GCCGAAGCCCTGTTCATTA	19	
OmpC	OmpC-F3	AGGGTTAATCAGTAAGCAGTGG	22	1090/This study
	OmpC-R3	AGGGTGAATTGTAAGAACCGAA	22	
OmpA	OmpA-F2	GGATGATAACGAGGCGCAAA	20	1196/ This study
	OmpA-R2	CAACCAGATGTCTACGCTGAAG	22	
OmpX	OmpX-F1	GGATTTACTTGAAGCACATTTGA GG	25	~500/ This study
	OmpX-R1	CCGAAGTGATTAGAAGCGGTAA	22	
FhuA	FhuA-F3	TCTTCCGTGACGCTTCATTC	20	~2100/This study
	FhuA-R3	ACAACGTACCCTGGCAAATAA	21	
LamB	LamB-F3	CCCAGACGCTTTACCAGATT	20	419/ This study
	LamB-R3	GGCCTTCTGGCATCTCTTTAT	21	

Table S4: Screening of Virulence factors in environmental & clinical *Enterobacter* isolates

	Strains tested	Hemagglutination assay (Fimbriae)	Serum resistance	Biofilm formation
Environmental	SR4.9	Positive	Grade 5	Weakly adherent
	DL4.3	Positive	Grade-2	Weakly adherent
	DL4.6	Late Positive	Grade-2	Weakly adherent
	SR5.7	Positive	Grade-2	Strongly adherent
	DL5.1	Positive	Grade-3	Moderately adherent
Clinical	EcTATAH41	Positive	Grade 5	Strongly adherent
	EspIMS6	Positive	Grade 5	Strongly adherent
	EcIMS18	Positive	Grade 5	Weakly adherent
	EcIMS21	Positive	Grade-3	Strongly adherent
	EspAH2	Positive	Grade-2	Moderately adherent
	EspAH3	Late Positive	Grade-2	Strongly adherent
	EspAH4	Positive	Grade-3	Strongly adherent
	E. cloacae 13047	Positive	Grade-5	Strongly adherent

Note: Hemagglutination test for presence of type-1 fimbriae, Serum resistance and biofilm formation ability was assessed for selected MDR *Enterobacter* isolates.

Table S5: Criteria for determination of grades of serum resistance (Sahly et al., 2004)

Grade	Viable counts	Viable counts	Interpretation
**1**	< 10% after 1 and 2 hours	<0-1% after 3 hours	Highly sensitive
**2**	10-100% after 1 hour	< 10% after 3 hours	
**3**	> 100 % after 1 hour	< 100% after 2 and 3 hour	Intermediately susceptible
**4**	> 100 % after 1 and 2 hours	< 100% after 3 hour	
**5**	>100% after 1, 2 and 3 hour	fell at some time during the 3 h period	Resistant
**6**	>100% after 1, 2 and 3 hour	Rise in cell count throughout 3 h	

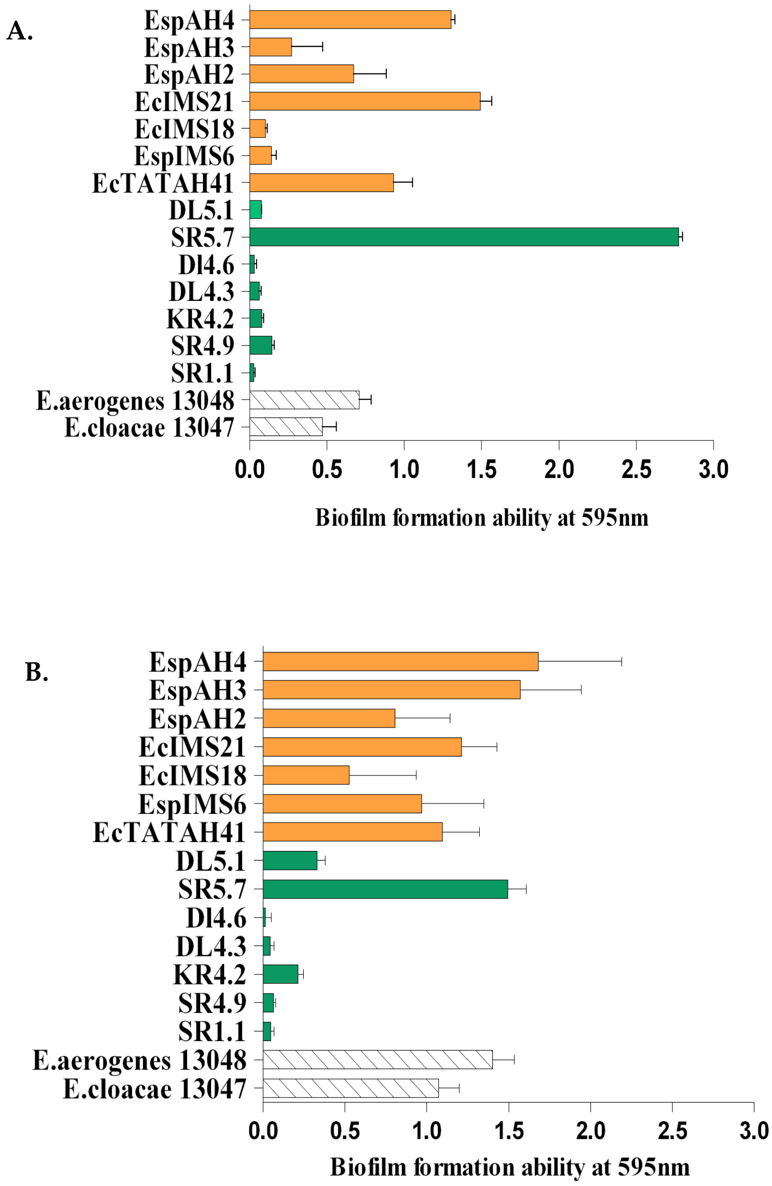

Figure S1: Biofilm formation of *Enterobacter* isolates by crystal violet staining method at 24 hours (A) and 48 hours (B). The X-axis represented the O.D. value of bound crystal violet at 595 nm. The Y-axis represented the *Enterobacter* isolates being tested. The orange bar and green bar in the graph indicated clinical and environmental *Enterobacter* isolates respectively, whereas white bar represented the ATCC *Enterobacter* type strains: ATCC 13047 and ATCC 13048.

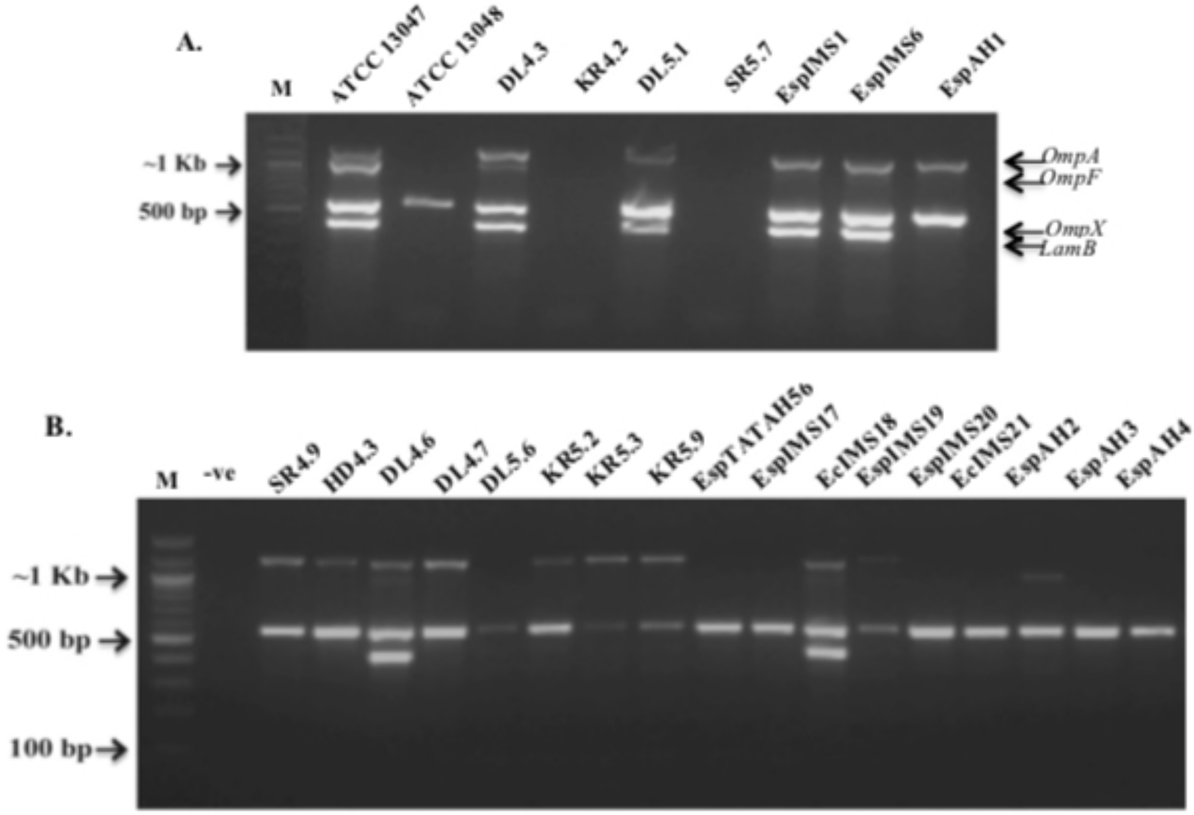

Figure S2: Agarose gel (1.5%) images of multiplex PCR products: Multiplex PCR products resulted in four distinct bands of OMPs in *E. cloacae* ATCC 13047, *OmpA* (1196 bp), *OmpF* (1001 bp), *OmpX* (~500 bp) and *LamB* (419 bp). Taking ATCC 13047 as control, hexaplex PCR based screening was performed (as shown in A & B) with other clinical and environmental *Enterobacter* isolates.

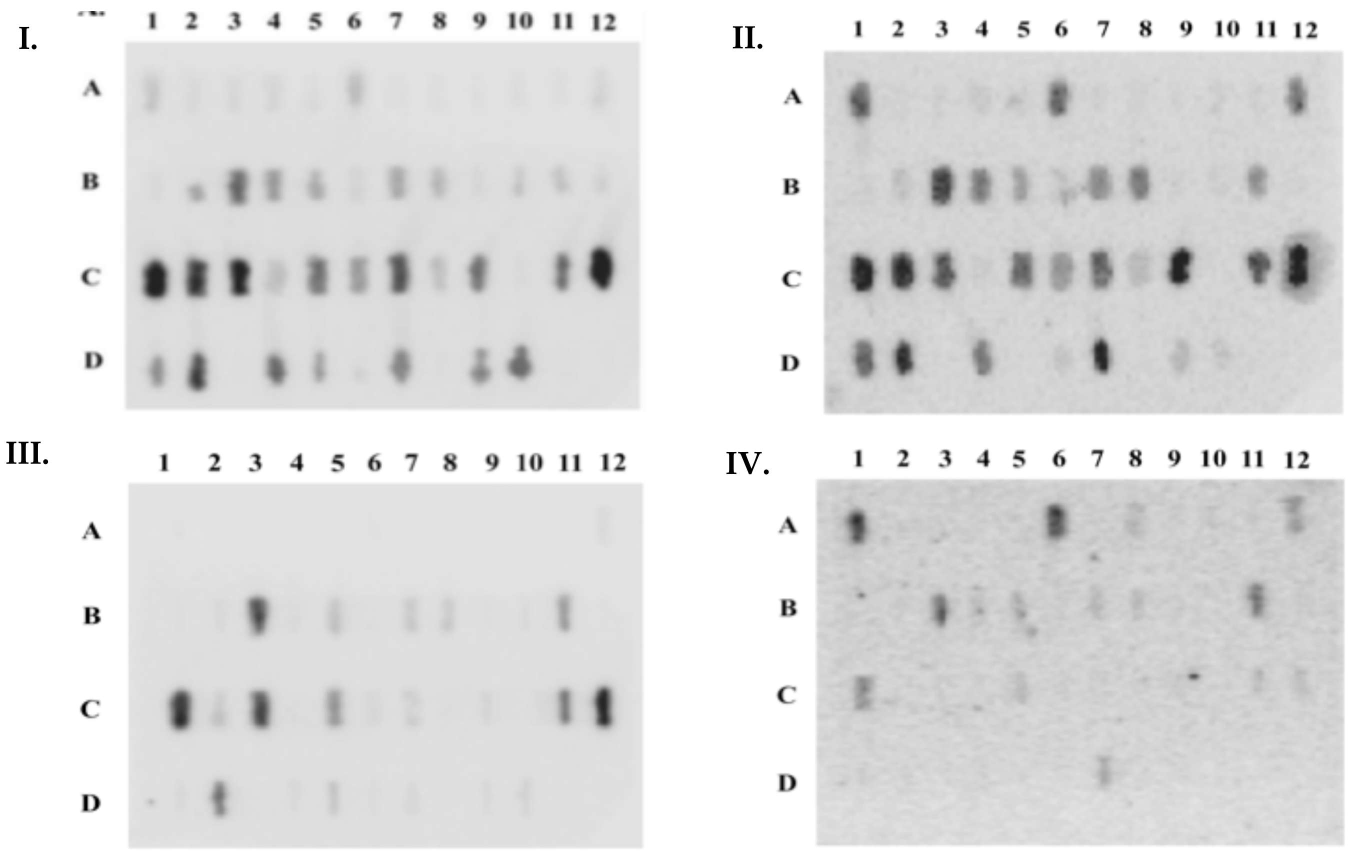

	1	2	3	4	5	6	7	8	9	10	11	12
A	EcTATAH41	EspTATAH56	EspIMS1	EspIMS4	EspIMS5	EspIMS6	EspIMS7	EspIMS8	EspIMS9	EspIMS10	EspIMS11	EspIMS13
B	EspIMS16	EspIMS17	EcIMS18	EspIMS19	EspIMS20	EcIMS21	EspAH1	EspAH2	EspAH3	EspAH4	ATCC13047	ATCC13048
C	SR1.1	SR1.5	HD1.9	KR1.8	SR2.2	SR2.3	SR2.4	HD2.1	SR4.9	KR4.2	DL4.3	DL4.6
D	DL4.7	HD4.3	SR5.7	KR5.2	KR5.3	KR5.9	DL5.1	DL5.6	ATCC13883	ATCC25922	ATCC27853	

Figure S3: Slot Blot images of OmpA (I), OmpX (II), OmpF (III) and LamB (IV)- Panel **A** and **B** contained clinical *Enterobacter* isolates whereas panel **C** and **D** contained environmental *Enterobacter* isolates (Except for positions-B11, B12, D9, D10 and D11 which contained ATCC type strain and D12 remained blank). The detailed order of genomic DNA loading in the slot blot is mentioned in the table placed below the figure.

Table S6: Correlation matrix demonstrating association between OMPs and in-vitro adhesion and invasion frequency in *Enterobacter* isolates as measured using XLSTAT software. Values in bold are different from 0 with a significance level alpha=0.05.

Variables	OmpA	OmpX	OmpF	LamB	Adhesion frequency	Invasion frequency
**OmpA**	***1***					
**OmpX**	***0.810***	***1***				
**OmpF**	*0.289*	*-0.134*	***1***			
**LamB**	***0.671***	***0.828***	*-0.043*	***1***		
**Adhesion frequency**	*0.327*	*0.404*	*0.523*	*0.269*	***1***	
**Invasion frequency**	*0.259*	*0.318*	***0.615***	*0.381*	***0.934***	***1***

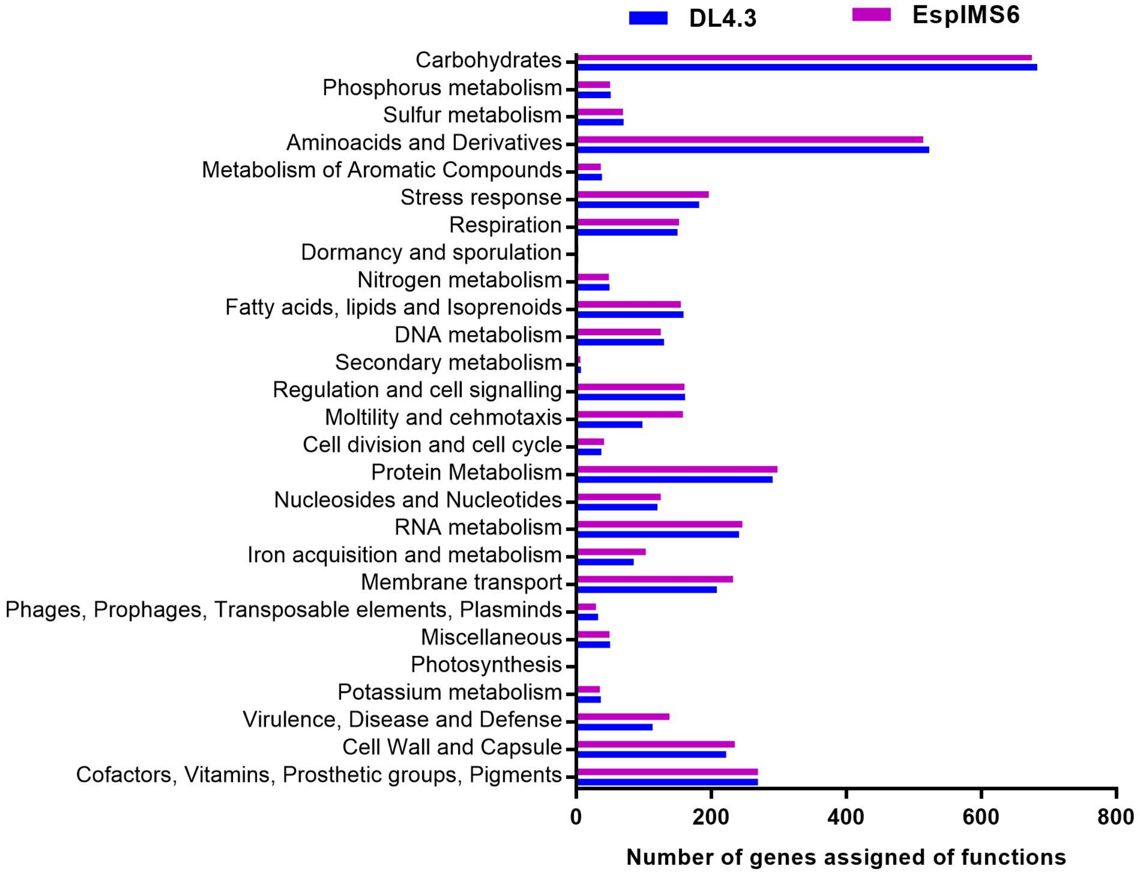

Figure S4: Comparative overview of number of genes involved in different subcategories and subsytems associated with functions in environmental isolate (DL4.3) and clinical isolate(EspIMS6)
